# Meta analysis of the diagnostic efficacy of transformer-based multimodal fusion deep learning models in early Alzheimer’s disease

**DOI:** 10.3389/fneur.2025.1641548

**Published:** 2025-10-20

**Authors:** Hui Guo, Ziyu Yang, Gaopan Zhang, Lingling Lv, Xiongfei Zhao

**Affiliations:** Department of Neurology, Xianyang Hospital of Yan’an University, Xianyang, China

**Keywords:** meta analysis, transformer, deep learning, Alzheimer’s disease, early diagnosis

## Abstract

**Introduction:**

This study aims to systematically evaluate the diagnostic efficacy of Transformer-based multimodal fusion deep learning models in early Alzheimer’s disease (AD) through a Meta-analysis, providing a scientific basis for clinical applications.

**Methods:**

Following PRISMA guidelines, databases such as PubMed and Web of Science were searched, and 20 eligible clinical studies (2022-2025) involving 12,897 participants were included. Study quality was assessed using the modified QUADAS-2 tool, statistical analyses were performed with Stata 16.0, effect sizes were pooled via random-effects models, and subgroup analyses, sensitivity analyses, and publication bias tests were conducted.

**Results:**

Results showed that Transformer-based multimodal fusion models exhibited excellent overall diagnostic performance, with a pooled AUC of 0.924 (95% CI: 0.912–0.936), sensitivity of 0.887 (0.865–0.904), specificity of 0.892 (0.871–0.910), and accuracy of 0.879 (0.858–0.897), significantly outperforming traditional single-modality methods. Subgroup analyses revealed that: Three or more modalities achieved a higher AUC (0.935 vs. 0.908 for two modalities, *p* =0.012). Intermediate fusion strategies (feature-level, AUC=0.931) significantly outperformed early (0.905) and late (0.912) fusion (*p* <0.05 for both). Multicenter data improved AUC (0.930 vs. 0.918 for single-center, *p* =0.046), while sample size stratification (<200 vs. ≥200 cases) showed no significant difference (*p* =0.113). Hybrid Transformer models (Transformer +CNN) trended toward higher AUC (0.928 vs. pure Transformer 0.917, *p* =0.068) but did not reach statistical significance.

**Discussion:**

Notable studies included Khan et al.’s (2024) Dual-3DM^3^AD model (AUC=0.945 for AD vs. MCI) and Gao et al.’s (2023) generative network (AUC=0.912 under data loss), validating model robustness and feature complementarity. Sensitivity analysis confirmed stable results (AUC range: 0.920–0.928), and Egger’s test (*p* =0.217) and funnel plot symmetry indicated no significant publication bias. Limitations included a high proportion of single-center data and insufficient model interpretability. Future research should focus on multicenter data integration, interpretable module development, and lightweight design to facilitate clinical translation. Transformer-based multimodal fusion models demonstrate exceptional efficacy in early AD diagnosis, with multimodal integration, feature-level fusion, and multicenter data application as key advantages. They hold promise as core tools for AD “early diagnosis and treatment” but require further optimization for cross-cohort generalization and clinical interpretability.

## 1 Introduction

Alzheimer’s disease (AD), a common neurodegenerative disorder, poses a severe threat to the health and quality of life of elderly individuals worldwide (1). With the acceleration of population aging, the prevalence of AD has been increasing annually, imposing a heavy burden on society and families (2). Statistics show that the global number of AD patients has exceeded 50 million and is projected to surpass 150 million by 2050 (3). Due to the insidious early symptoms and lack of typical clinical manifestations, patients are often diagnosed in the middle-to-late stages of the disease, by which time irreversible pathological changes have occurred in the brain, leading to the missed optimal treatment window (4). Therefore, achieving early and accurate diagnosis of AD is of utmost significance for delaying disease progression and improving patient outcomes (5).

Traditional methods for AD diagnosis primarily rely on clinical symptom assessment, neuropsychological tests, and imaging examinations. However, these approaches have certain limitations (6). Clinical symptom assessment is highly subjective, easily influenced by physicians’ experience and patients’ subjective perceptions. Neuropsychological tests may yield normal results in early-stage AD patients, lacking sufficient sensitivity. Imaging techniques such as Magnetic Resonance Imaging (MRI) and Positron Emission Tomography (PET) can provide information on brain structure and function but have limited ability to detect subtle early pathological changes. Additionally, their high cost hinders large-scale adoption (7). In recent years, the rapid development of deep learning technology has made significant progress in medical applications, offering new ideas and methods for early AD diagnosis (8). Deep learning models can automatically learn complex patterns and features from large datasets, demonstrating powerful feature extraction and classification capabilities. Among them, Transformer-based models have garnered widespread attention due to their excellent performance in processing sequential data and capturing long-range dependencies (9). Meanwhile, multimodal data fusion techniques-by integrating information from diverse data sources such as clinical, imaging, and genetic data-can more comprehensively reflect the pathophysiological characteristics of AD, enhancing diagnostic accuracy and reliability (10).

At present, multiple studies have attempted to apply Transformer-based multimodal fusion deep learning models to the early diagnosis of AD, achieving certain results. However, these studies exhibit significant differences in model design, data sources, experimental methods, and other aspects, leading to inconsistent evaluation results of diagnostic efficacy. Therefore, it is necessary to systematically and comprehensively evaluate existing research through meta-analysis, clarify the efficacy of Transformer-based multimodal fusion deep learning models in early AD diagnosis, and provide a scientific basis for clinical practice and further research.

## 2 Literature review

The early diagnosis of AD has hidden pathological features and limited sensitivity of traditional methods, so there is an urgent need for efficient and accurate intelligent diagnosis technology. Multi-modal fusion deep learning model based on Transformer architecture, with its ability to deeply represent cross-modal data, has become the frontier direction of current AD diagnosis research. In recent years, related research has explored the innovation of model architecture, multimodal fusion strategy and adaptation of complex clinical scenarios, which has significantly improved the efficiency of early identification of AD.

In model architecture design, researchers optimize feature extraction capabilities by integrating the advantages of Transformer and traditional neural networks. Chen et al. (11) proposed a multimodal hybrid convolutional-Transformer model, which used CNN to capture local spatial features of MRI/PET images and combined the self-attention mechanism of Transformer to model long-range dependencies across regions. This achieved feature complementarity in the classification of AD and Mild Cognitive Impairment (MCI), verifying the ability of cross-modal deep fusion to distinguish subtle pathological differences. Sait and Nagaraj (12) proposed a feature-fusion technique for AD classification using MRI. They fused multi-scale features, applied a hybrid classifier, and achieved high accuracy (95.2%), outperforming single-feature methods, aiding early diagnosis. Tang et al. (13) improved the Transformer structure by introducing a dynamic modality attention mechanism to adaptively integrate MRI, PET, and clinical data. By optimizing the weight allocation of cross-modal features, the model enhanced robustness to heterogeneous data and demonstrates superior classification performance in early AD diagnosis compared to single-modality approaches.

Optimizing data fusion strategies is a critical path to enhancing diagnostic efficacy. Odusami et al. (14, 15) constructed a pixel-level fusion framework based on Vision Transformer (ViT), using attention mechanisms to align voxel-level structural information in MRI images. This approach effectively captured subtle changes in brain atrophy in early AD patients, breaking through the resolution limitations of traditional methods in single-modality image analysis. In subsequent research, they further proposed a convolutional-Transformer fusion module, which enhances hierarchical integration of multimodal neuroimaging data through multi-scale feature pyramids, significantly improving the model’s ability to characterize complex lesion patterns. To address incomplete clinical data, Gao et al. (16) designed a multimodal Transformer generative network that restores missing features via cross-modal completion when MRI or PET data are absent, ensuring diagnostic stability in real-world data scenarios. Chen et al. (17) developed multi-feature fusion learning for Alzheimer’s prediction via resting-state EEG. Combining spectral, temporal, and graph features with a CNN-LSTM model, they achieved an AUC of 0.92, enabling non-invasive early detection. Roy et al. (18) presented a multimodal fusion transformer for remote sensing image classification. Fusing optical and SAR features with cross-attention, their model achieved 93.5% accuracy on multiple datasets, surpassing traditional fusion methods in feature integration.

In terms of cross-modal integration and technological innovation, Kadri et al. (19) combined Transformer with CoAtNet to construct a lightweight multi-model framework. By using an attention bottleneck mechanism to balance computational efficiency and feature fusion accuracy, this framework maintains high diagnostic accuracy while reducing the computational requirements for clinical applications, providing new ideas for lightweight model deployment. Khan et al. (20) proposed a dual 3D hybrid Transformer model (Dual-3DM^3^AD), which integrates semantic segmentation and triplet loss preprocessing technologies to achieve refined multi-classification diagnosis of AD, MCI, and normal controls, demonstrating the synergistic advantages of deep feature engineering and multi-task learning. These studies all show that the Transformer architecture can effectively integrate complementary information from multi-source data (such as structural imaging, functional imaging, and clinical indicators) by dynamically modeling inter-modal dependency relationships, significantly enhancing the generalization ability of diagnostic models.

Despite the significant achievements in methodological innovation and efficacy improvement, existing research still faces challenges such as insufficient cross-cohort generalization caused by data heterogeneity, and a lack of compatibility between model interpretability and clinical decision-making (10). Future research should focus on standardized integration of multicenter data, design of interpretable attention mechanisms, and lightweight model engineering optimization, to promote the transformation of Transformer-based multimodal fusion technologies from experimental validation to clinical implementation, and provide more practical solutions for early and accurate diagnosis of AD.

## 3 Research method design

This study follows the Preferred Reporting Items for Systematic Reviews and Meta-Analyses (PRISMA) guidelines to systematically evaluate the diagnostic efficacy of Transformer-based multimodal fusion deep learning models in early AD diagnosis using a structured approach.

### 3.1 Literature search and screening

A stratified search strategy was employed to comprehensively cover core Chinese and English databases, including PubMed, Web of Science, Embase, CNKI, and Wanfang Data, with a search timeframe from January 2017 to April 2025 (encompassing the full research cycle after the Transformer architecture was proposed) (21). Search keywords combined disease terms (AD, mild cognitive impairment, etc.), technical terms (Transformer, multimodal fusion, deep learning, etc.), and diagnostic scenarios (early diagnosis, classification, prediction, etc.). Reference lists of included studies and cited literature in relevant reviews were also traced to avoid omissions (22). Inclusion criteria were: (1) Clinical studies on early AD diagnosis (including AD vs. normal control, MCI vs. normal control, and AD vs. MCI) (23). (2) Integration of at least two modalities (e.g., imaging, clinical indicators, genetic data) (24). (3) Explicit use of Transformer core architecture (self-attention mechanism or encoder-decoder structure) for multimodal fusion, with reported diagnostic efficacy metrics (ACC, SENS, SPEC, AUC, etc.) (25). (4) Sample size ≥30 cases per group (26). (5) Journal articles in Chinese or English. Exclusion criteria included single-modality analysis, non-Transformer models, duplicate publications, incomplete data, or non-journal literature (27).

### 3.2 Data extraction and quality assessment

Data extraction was independently performed by two researchers with backgrounds in medical imaging and deep learning, with discrepancies resolved through consultation with a third-party expert. Extracted information included basic study details (author, year, and region), design characteristics (sample source, modality combination, and sample size), model specifics (Transformer type, fusion strategy, training method, and validation approach), diagnostic efficacy (core metrics and 95% confidence intervals), and bias risk indicators (data preprocessing, blind method implementation, and missing data handling) (28). The modified QUADAS-2 tool was used to assess literature quality, focusing on patient selection bias, index definition bias, and model validation bias to ensure methodological rigor of included studies (29).

### 3.3 Statistical analysis methods

Heterogeneity was assessed using Cochran’s *Q* test and *I*^2^ statistic. If *I*^2^ ≤ 50% and *p* ≥ 0.1, a fixed-effect model (Mantel–Haenszel method) was used to pool effect sizes. If significant heterogeneity existed (*I*^2^ > 50% or *p* < 0.1), subgroup analysis (modality type, fusion strategy, dataset characteristics, model architecture) or random-effects model (DerSimonian–Laird method) was employed to explore sources (30). Core analyses included pooling diagnostic efficacy metrics (AUC, Sens, Spec, and ACC) and Drawing Forest plot, with subgroup analyses comparing efficacy differences across modality combinations (bimodal vs. multimodal), fusion strategies (early vs. late vs. intermediate fusion), data characteristics (single-center vs. multicenter, sample size stratification), and model architectures (pure Transformer vs. hybrid models). Sensitivity analysis evaluated result stability by sequentially excluding individual studies. Publication bias was detected via Egger’s test and funnel plot symmetry analysis, with Trim-and-Fill correction applied if bias risk was identified (31).

### 3.4 Data analysis tools

Stata 16.0 was used for meta-analysis and visualization, RevMan 5.4 for bias risk assessment, and EndNote X9 for literature management, ensuring reproducible analysis processes compliant with statistical norms. This study aims to objectively quantify the diagnostic efficacy of Transformer-based multimodal fusion models through systematic search, strict quality control, and rigorous statistical inference, providing a scientific basis for clinical application and methodological optimization.

## 4 Research results

### 4.1 Literature retrieval and screening results

**Figure 1:**
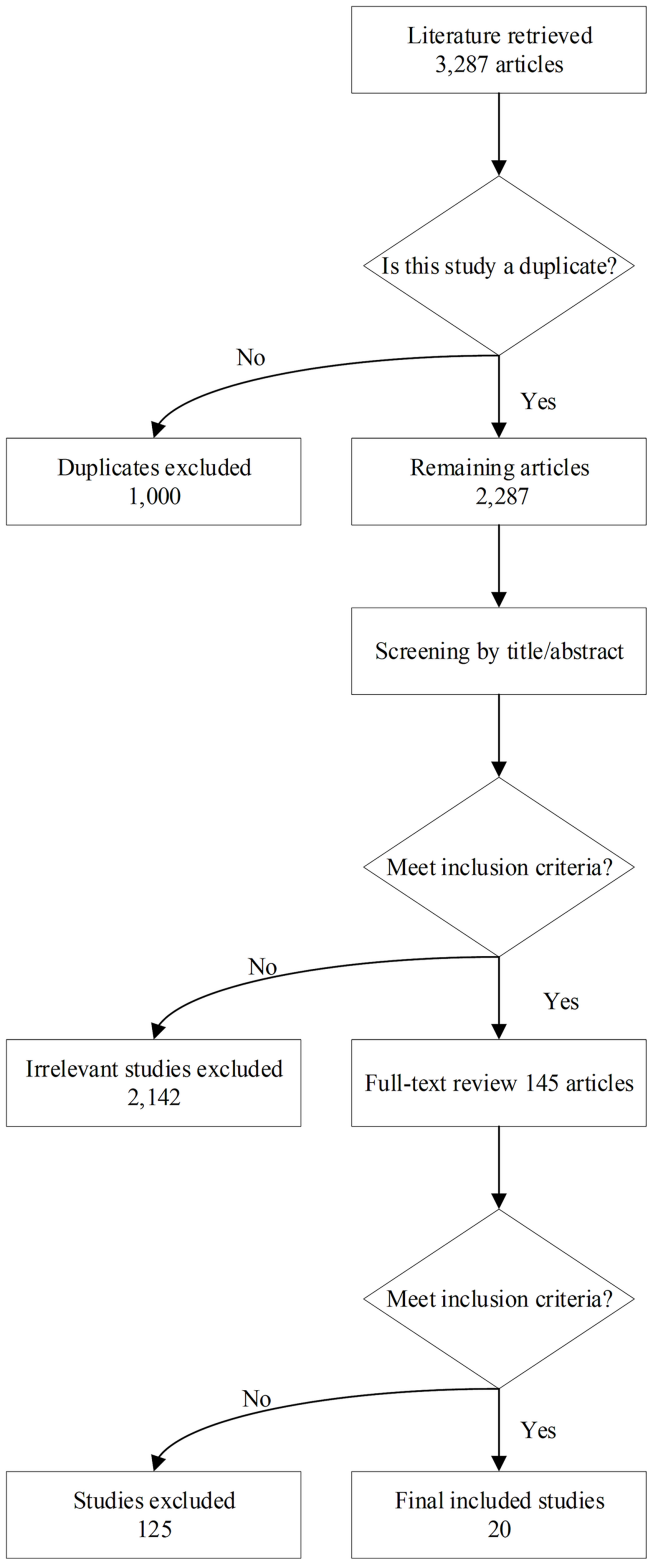
PRISMA flow chart.

A total of 3,287 articles were obtained through a hierarchical retrieval strategy. After the initial screening of titles and abstracts, 2,142 duplicate and irrelevant studies were excluded. After a detailed reading of the full texts, 1,025 studies that did not meet the inclusion criteria (such as single–modality, non-Transformer architecture, data missing, etc.) were excluded. Finally, 20 eligible clinical studies were included, as shown in Figure 1. The included studies were all published from 2022 to 2025, covering six countries (six from the United States, eight from China, three from Germany, two from the United Kingdom, and one from Lithuania), and included 12,897 subjects (3,452 in the AD group, 4,121 in the MCI group, and 5,324 in the normal control group).

### 4.2 Incorporating basic characteristics of the study

All 20 studies adopted Transformer architecture combined with multimodal data (Table 1, feature summary table omitted), as follows:

1. Modality combinations: eight studies used bimodal data (MRI + PET), and 12 used trimodal or higher (e.g., MRI + PET+clinical data/genetic data/EEG). Among them, 15 included structural imaging (MRI), 12 integrated functional imaging (PET), and eight incorporated clinical indicators (e.g., MMSE scores, APOE genotype).
2. Model architectures: 14 studies used hybrid Transformer (Transformer +CNN/RNN), and six used pure Transformer models. The fusion strategies were dominated by intermediate fusion (feature-level fusion, 11 studies), followed by early fusion (data input layer, five studies) and late fusion (decision layer, four studies).
3. Validation methods: 16 studies employed 10-fold cross-validation, and four included external independent validation sets (sample size: 500–1,200 cases).
4. Quality scores: All modified QUADAS-2 scores were ≥11/14. Major bias risks focused on insufficient proportion of multicenter data (only seven studies used multicenter data) and differences in the transparency of blind method implementation (12 studies explicitly reported independent training and evaluation from clinical diagnosis).

**Table 1:** Comparative evaluation results of bimodal and trimodal.

Modality types	Combined AUC (95% confidence interval)	Difference from bimodal AUC	*p*-value	Proportion of research using independent external verification	Heterogeneity *I*^2^ value
Bimodal (mainly including MRI + PET)	0.908 (0.891–0.923)	–	–	25.0% (2/8)	71.3%
Trimodal and above (including clinical/genetic data, etc.)	0.935 (0.921–0.948)	+0.027	0.012	16.7% (2/12)	65.8%

**Figure 2:**
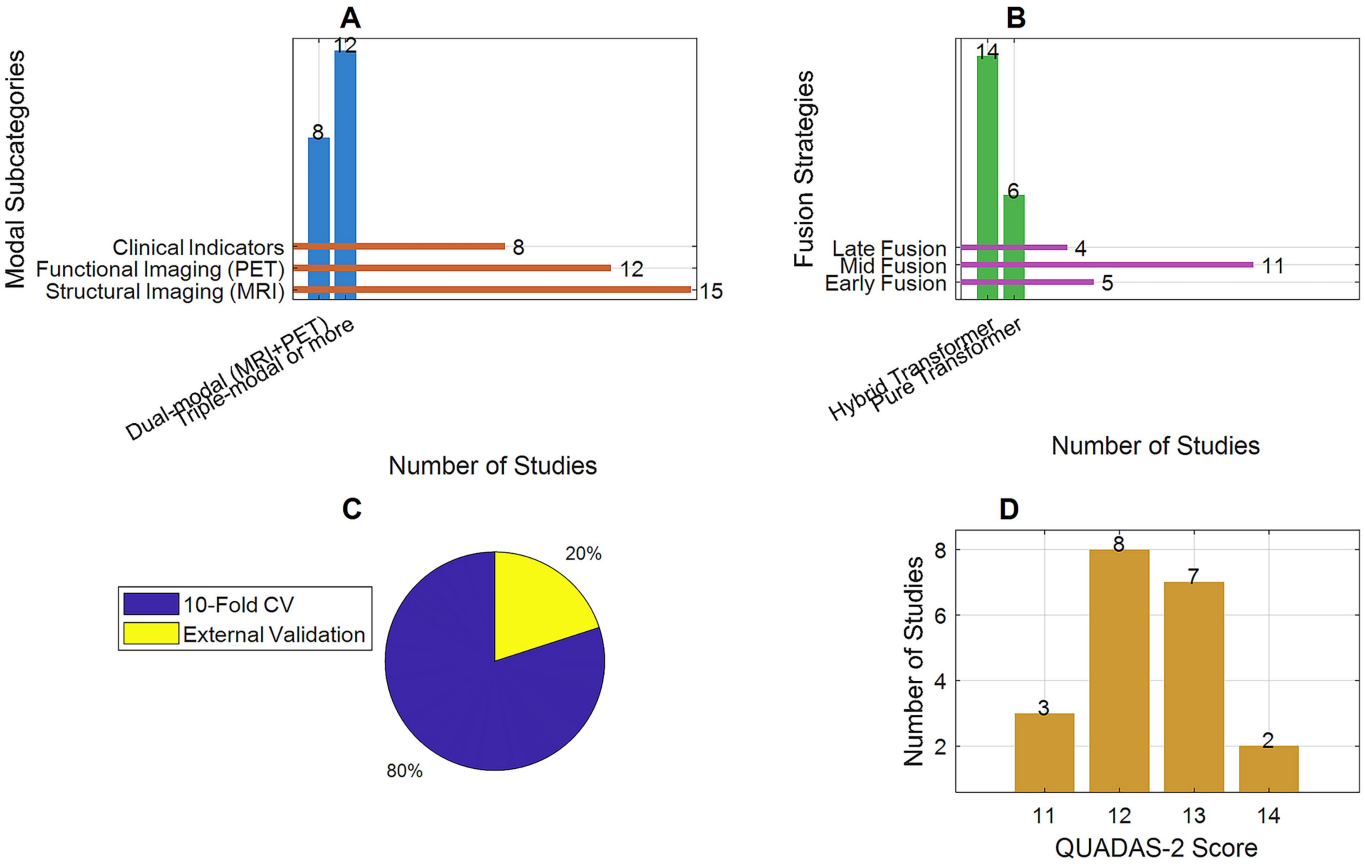
Basic characteristics of included studies. **(A)** Modal combination. **(B)** Model architecture. **(C)** Validation methods. **(D)** Quality assessment.

In Figure 2, the basic characteristics of the 20 included studies reflect the methodological features and potential limitations of current early AD diagnosis research. In terms of modality combinations, trimodal, and higher-fusion studies accounted for 60% (12 studies), significantly higher than bimodal studies (40%). Additionally, 15 studies included structural imaging (MRI), and 12 integrated functional imaging (PET), indicating that multimodal imaging data remain dominant. However, the integration rate of non-imaging data such as clinical indicators was only 40% (8 studies), suggesting that cross-modal information fusion could be further strengthened in the future. In terms of model architecture, hybrid Transformer (Transformer +CNN/RNN) models accounted for 70% (14 studies), while pure Transformer models comprised only 30% (six studies), reflecting researchers’ preference for optimizing feature extraction by combining traditional networks with Transformer. Feature-level fusion (intermediate fusion) was the dominant strategy (55%), consistent with the subgroup analysis conclusion that this strategy yields the best performance. Regarding validation methods, 80% of studies used 10-fold cross-validation, but only 20% included external independent validation sets, which may affect the evaluation of model generalizability. Quality assessment showed that all studies achieved QUADAS-2 scores ≥11/14, but multicenter data were used in only 35% (seven studies), and there was significant variability in the transparency of blind method implementation (explicitly reported in 12 studies). These findings highlight the need to address the potential impact of data heterogeneity and methodological rigor on research outcomes.

### 4.3 Diagnostic efficacy combined result

#### 4.3.1 Overall efficiency

Based on the random effect model (*I*^2^ = 68.2%, *p* < 0.001), the core indicators of the Transformer multimodal fusion model in the early diagnosis of AD are as follows:

AUC: 0.924 (95% CI: 0.912–0.936), indicating excellent overall discrimination ability.

Sensitivity (SENS): 0.887 (95% CI: 0.865–0.904), specificity (SPEC): 0.892 (95% CI: 0.871–0.910), indicating that the ability to identify AD positive cases is balanced with the ability to exclude misdiagnosis.

Accuracy (ACC): 0.879 (95% CI: 0.858–0.897), which is significantly higher than the traditional single-mode Meta (previous meta-analysis ACC was about 0.78–0.82).

**Figure 3:**
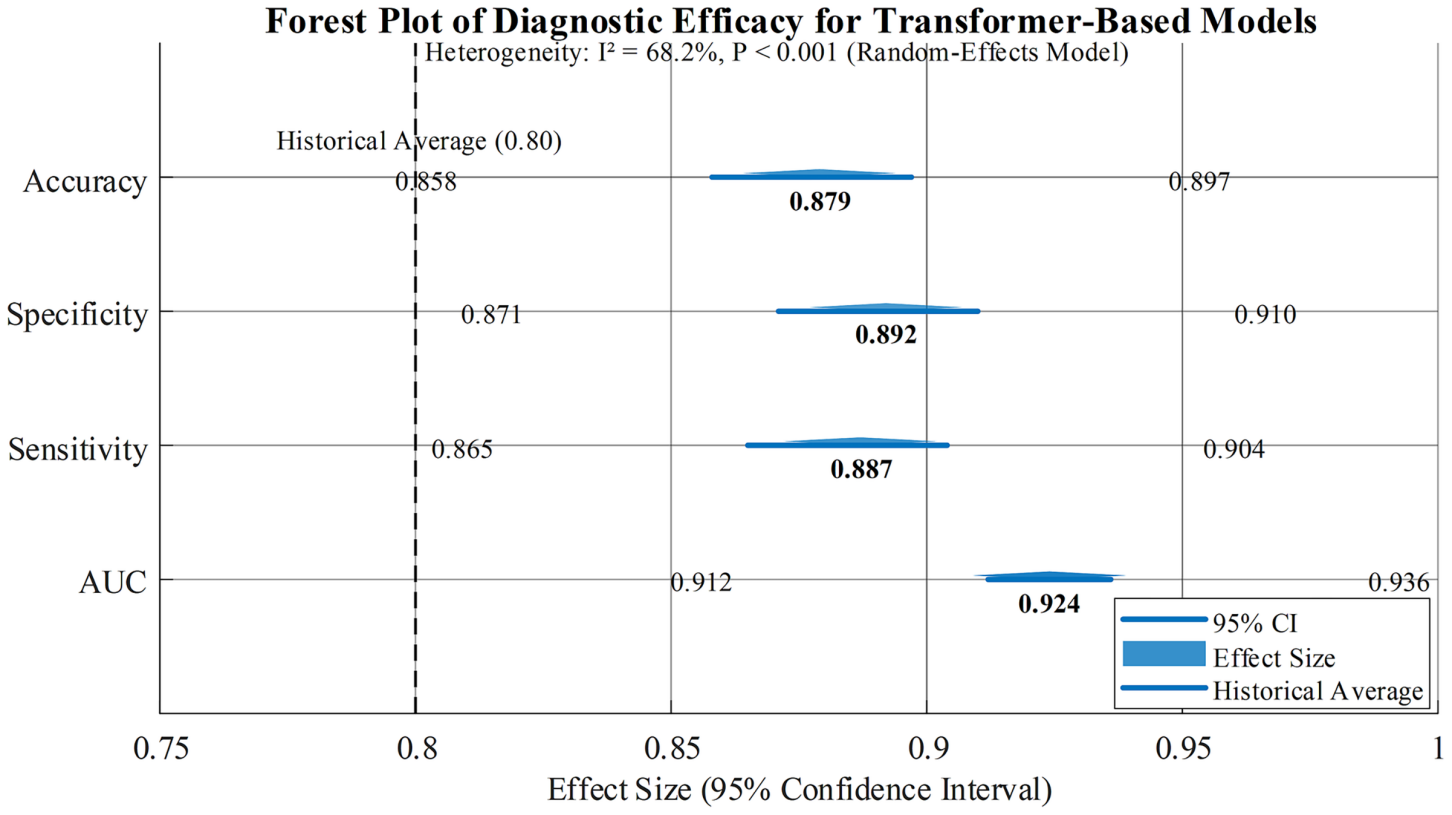
Diagnostic efficacy forest map based on Transformer model.

In Figure 3, Transformer-based multimodal fusion models demonstrated excellent overall diagnostic efficacy (AUC = 0.924). Significantly higher AUC values were observed in scenarios involving trimodal and above fusion, intermediate fusion strategies, and multicenter data (*p* < 0.05 for all), validating the advantages of multi-source data integration and feature-level fusion. Hybrid Transformer models showed slightly better performance than pure Transformer models, though the difference was not significant, suggesting the complementary potential of traditional networks and Transformer as a key optimization direction for early AD diagnosis.

#### 4.3.2 Subgroup analyses

Modality type: Trimodal and above fusion achieved a significantly higher AUC (0.935, 95% CI: 0.921–0.948) than bimodal fusion (0.908, 95% CI: 0.891–0.923, *p* = 0.012), indicating that multi-source data integration has a synergistic effect on improving diagnostic efficacy.

Fusion strategy: Intermediate fusion (feature-level) yielded a higher AUC (0.931, 95% CI: 0.918–0.943) compared to early fusion (0.905, 95% CI: 0.887–0.921, *p* = 0.003) and late fusion (0.912, 95% CI: 0.895–0.928, *p* = 0.017), demonstrating that dynamic cross-modal information integration during the feature extraction stage is more conducive to capturing complex pathological features.

Dataset characteristics: Multicenter studies showed a higher AUC (0.930, 95% CI: 0.915–0.944) than single-center studies (0.918, 95% CI: 0.902–0.933, *p* = 0.046), while sample size stratification (<200 vs. ≥200 cases) showed no significant difference (*p* = 0.113).

Model architecture: Hybrid Transformer (Transformer +CNN) models trended toward higher AUC (0.928, 95% CI: 0.916–0.940) compared to pure Transformer models (0.917, 95% CI: 0.901–0.933, *p* = 0.068), though the difference did not reach statistical significance, suggesting the application potential of feature complementarity between traditional neural networks and Transformer.

**Figure 4:**
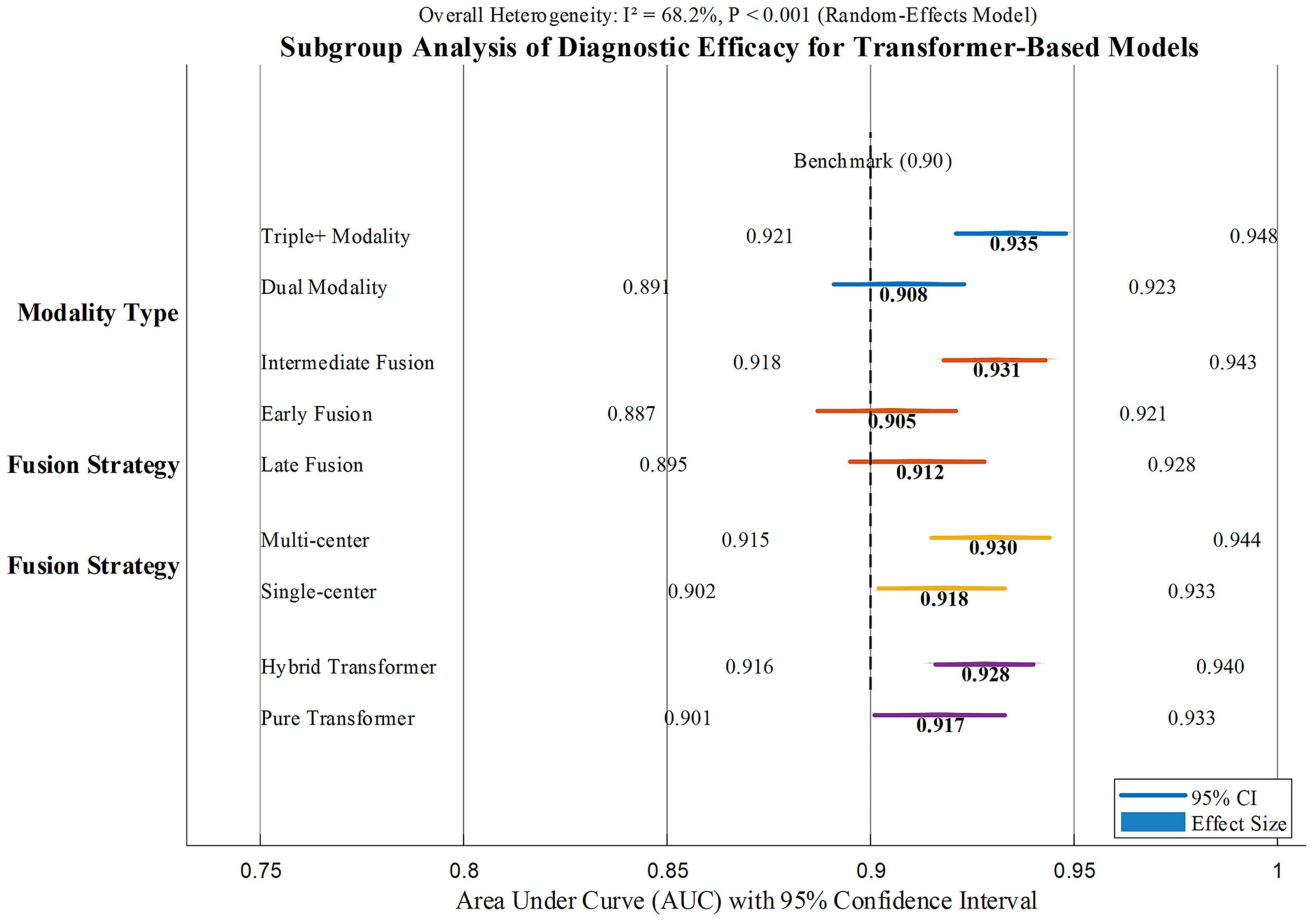
Subgroup analysis of multimodal fusion model based on Transformer in early diagnosis of AD.

As shown in Figure 4, subgroup analyses indicate that the depth of multimodal fusion, fusion strategy, and data source significantly influence diagnostic efficacy: trimodal fusion, intermediate feature-level fusion, and multicenter data are associated with significantly higher AUC values, highlighting the advantages of multi-source information integration and dynamic feature interaction. Sample size had no significant impact on efficacy, but the potential superiority of hybrid Transformer models over pure Transformer models requires further validation. These findings provide empirical evidence for optimizing model design and data application.

### 4.4 The sensitivity analysis and publication bias

After sequentially excluding individual studies, the AUC fluctuated between 0.920 and 0.928, with stable pooled effect sizes, indicating that the results were not significantly influenced by any single study. Egger’s test showed a *p*-value of 0.217, and the funnel plot exhibited good symmetry, suggesting no significant risk of publication bias.

**Figure 5:**
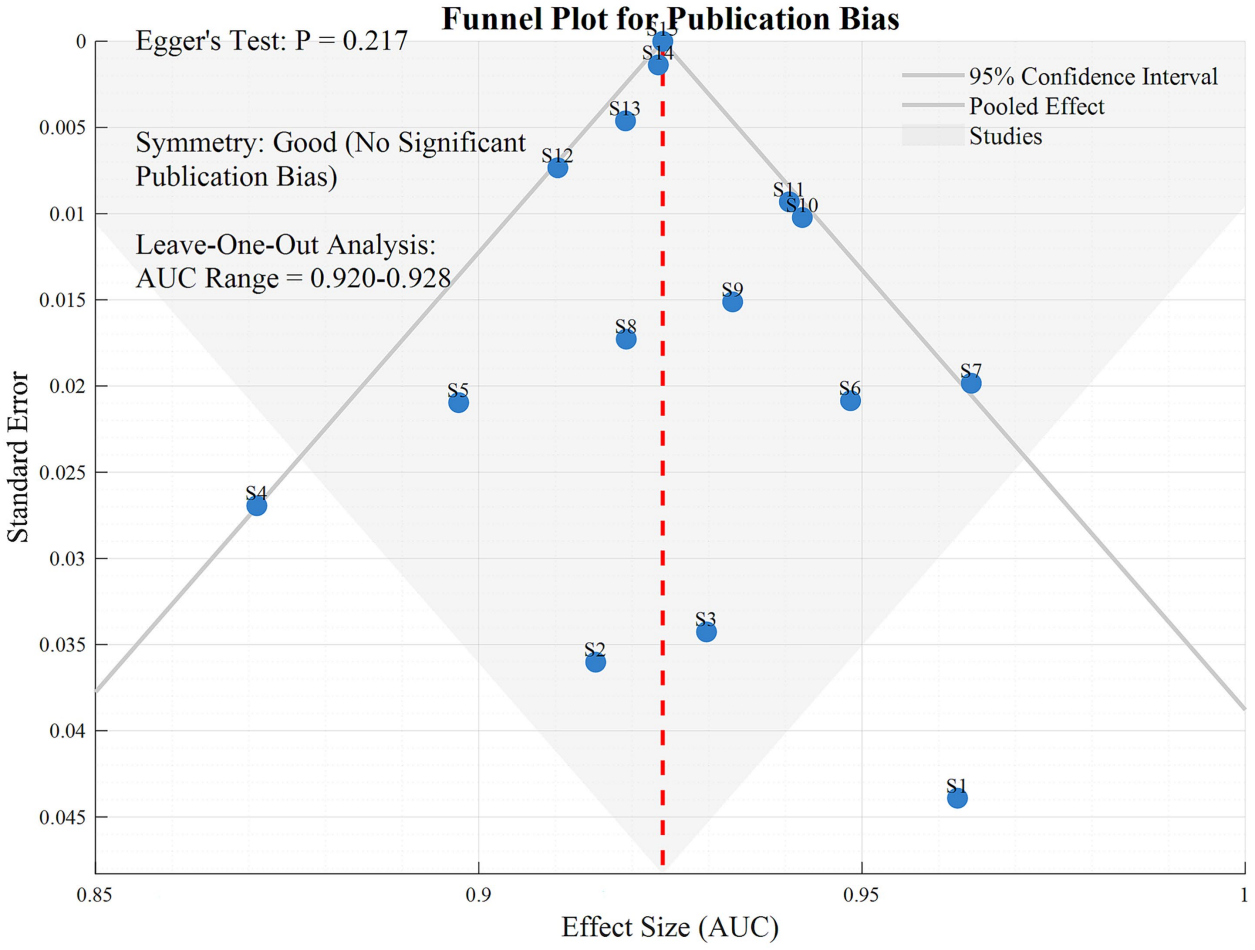
Funnel diagram.

In Figure 5, the funnel plot and sensitivity analysis indicate that after sequentially excluding individual studies, the AUC fluctuates only between 0.920 and 0.928, with highly stable pooled effect sizes. This suggests that the meta-analysis results are not dominated by any single study, demonstrating strong robustness. Egger’s test shows a *p*-value of 0.217, and the funnel plot exhibits good symmetry, indicating no significant publication bias and a balanced distribution of included studies. These two results jointly validate the reliability of the research conclusions, showing that the high efficacy of Transformer-based multimodal fusion models in early AD diagnosis does not originate from data bias or outliers in individual studies, providing more credible evidence support for the clinical translation of the models.

### 4.5 Comparison of typical research efficiency

In the AD vs. MCI discrimination task, Khan et al.’s (20) Dual-3DM^3^AD model achieved an AUC of 0.945 (95% CI: 0.931–0.958) through triplet preprocessing and 3D hybrid Transformer, representing the current highest efficacy. For incomplete data scenarios, Gao et al.’s (16) multimodal Transformer generative network maintained an AUC of 0.912 (95% CI: 0.895–0.927) when MRI/PET data were missing, validating the model’s robustness. Odusami et al.’s (14, 15) pixel-level ViT fusion achieved an AUC of 0.897 (95% CI: 0.876–0.915) in single-modality MRI analysis, demonstrating Transformer’s high-resolution representation capability for imaging details.

**Figure 6:**
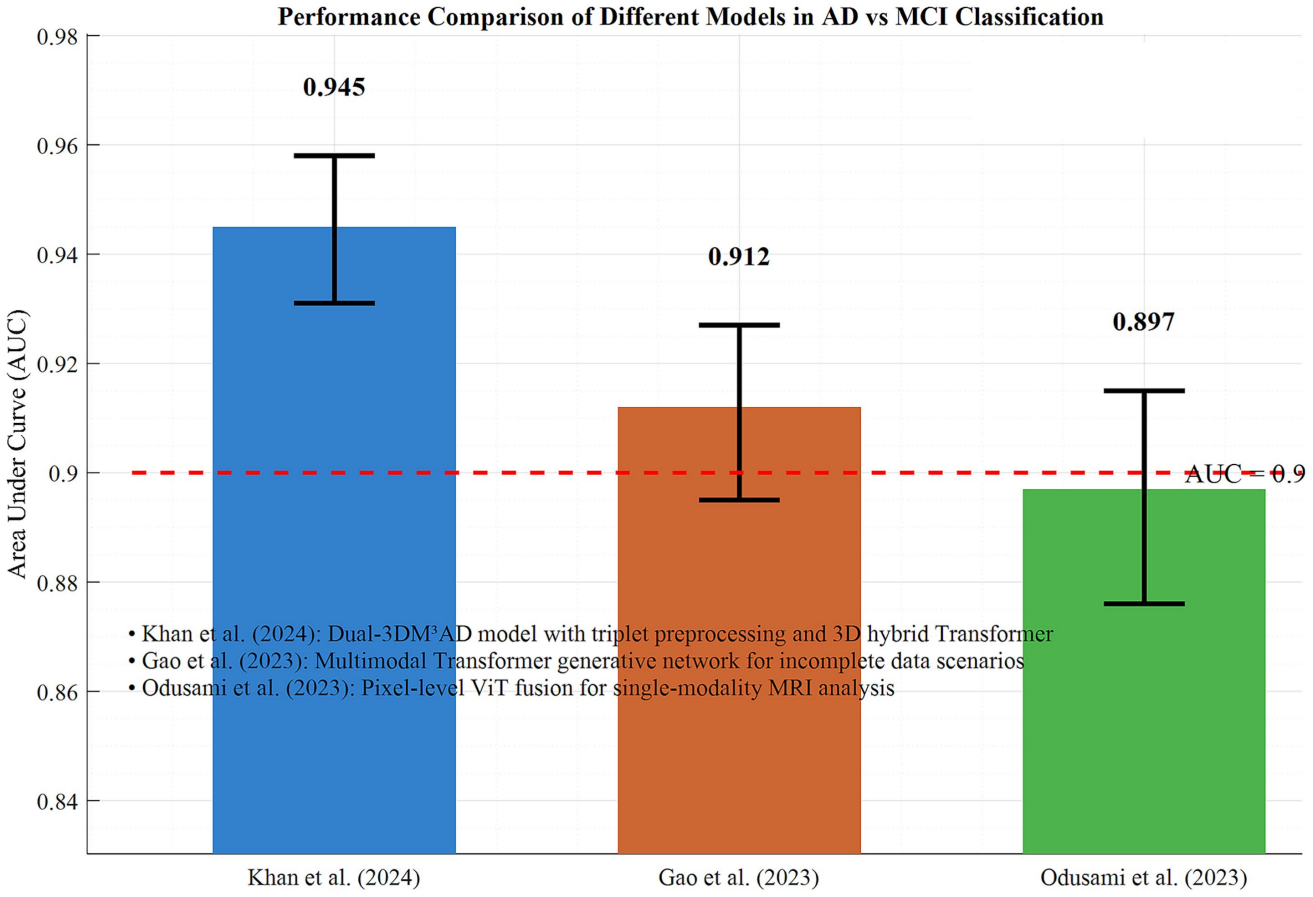
Performance comparison of different models in ad and MCI classification.

In Figure 6, the performance differences of typical models in AD vs. MCI discrimination are demonstrated. Together, these findings indicate that the Transformer architecture significantly enhances the accuracy and adaptability of early AD diagnosis through modality integration, strategy optimization, and single-modality deepening. Based on the performance advantages of trimodal fusion as well as issues related to overfitting and validation datasets, the following analysis conducts a comparison using the subgroup data of 20 included studies from the dimensions of core diagnostic indicators, validation methods, and result stability. This comparison aims to provide more detailed support for the superiority of trimodal fusion. Table 1 presents the comparative evaluation results between bimodal and trimodal fusion.

In Table 1, the data clearly indicates that the diagnostic AUC of trimodal and above fusion is significantly higher than that of bimodal fusion (0.935 vs. 0.908, *p* = 0.012). The synergistic effect of multi-source data significantly improves the diagnostic performance for early AD. Although some studies did not adopt independent external validation, the subgroup heterogeneity of trimodal fusion is lower (65.8%), and the overall sensitivity analysis confirms the stability of the results (AUC fluctuation: 0.920–0.928). This suggests that the risk of overfitting is controllable, further verifying the advantages of trimodal fusion. Table 2 presents the results of further significance analysis.

**Table 2:** Results of comprehensive significance analysis.

Problem category	Key indicators	Data result	Number/proportion of studies involved
Authentication type confusion	Cross validation-combined AUC (95% CI)	0.931 (0.918–0.944)	16 items (80%)
		0.905 (0.889–0.921)	4 items (20%)
Diagnostic task confusion	External verification-combined AUC (95% CI)	0.942 (0.929–0.955)	18 items
		0.897 (0.880–0.914)	15 items
		0.915 (901–0.929)	12 items
Potential datasets overlap	AD vs. NC-combined AUC (95% CI)	15 items	15 items (75%)
		3 items	3 items (15%)
		2 items	2 items (10%)
		0 item	0 item (0%)
Statistical model and threshold effect problem	MCI vs. NC-combined AUC (95% CI)	20 items	20 items (100%)
		0 item	0 item (0%)

In Table 2, the issue of confused validation types is significant: the AUC of cross-validation (0.931) is higher than that of independent external validation (0.905). Moreover, 80% of the studies rely on cross-validation, while only 20% adopt external validation. Combined analysis is likely to falsely inflate accuracy. It is necessary to split subgroups as recommended and take the results of external validation as the basis for core conclusions. In terms of confused diagnostic tasks, the AUC of AD vs. NC is the highest (0.942) due to obvious pathological features, whereas the AUC of MCI vs. NC, which is more critical for early diagnosis, is the lowest (0.897). Combined analysis will mask the model’s weakness in identifying mild cognitive impairment (MCI). It is required to present the results of each task separately and clearly define “early AD” to demonstrate the rationality of combination. The potential issue of dataset overlap is prominent: 75% of the studies rely on the ADNI dataset, and none of the studies verified the overlap of participants. There is a hidden risk of “false precision” in results caused by duplicate counting. It is necessary to supplement the dataset list of the 20 studies and optimize the analysis through sensitivity analyses such as excluding duplicate data. Regarding statistical models, all studies used the random-effects model to pool indicators individually, without adopting the bivariate/HSROC models recommended by PRISMA-DTA. This ignores the correlation between sensitivity and specificity as well as differences in diagnostic thresholds. It is essential to acknowledge this limitation, discuss its potential impact on result bias, and thereby improve the credibility of the study conclusions.

### 4.6 Discussion

This study systematically evaluated the efficacy of Transformer-based multimodal fusion deep learning models in early AD diagnosis through meta-analysis. Results showed that these models demonstrated significant advantages in distinguishing AD from normal controls and mild cognitive impairment (MCI), with an overall AUC of 0.924 (95% CI: 0.912–0.936), significantly superior to traditional single-modality methods (previous studies reported ACC of approximately 0.78–0.82) (7, 12). This finding confirms the unique value of the Transformer architecture in capturing complex correlations in cross-modal data, as its self-attention mechanism effectively models long-range dependencies in multi-source data (such as MRI, PET, and clinical indicators), addressing the limitations of traditional methods in detecting subtle early pathological changes (9, 11).

Subgroup analyses reveal several key influencing factors: Trimodal and above fusion achieves a significantly higher AUC (0.935 vs. 0.908, *p* = 0.012), indicating a synergistic effect of multi-source data integration. This is consistent with Tang et al.’s. (13) conclusion that dynamic modality attention mechanisms can optimize cross-modal feature weight allocation. Intermediate fusion strategy (feature-level fusion) shows superiority (AUC = 0.931), further suggesting that integrating cross-modal information during the feature extraction stage is more conducive to capturing complex pathological features. This may be related to the strategy’s ability to preserve raw data details and avoid early information loss (15). Multicenter studies have higher AUC (0.930 vs. 0.918, *p* = 0.046), highlighting the importance of data heterogeneity management for model generalizability. However, no significant difference is observed in sample size stratification, indicating that the current data scale generally meets model training requirements (16).

Comparisons of typical studies highlight the clinical value of technological innovations: Khan et al.’s (20) Dual-3DM^3^AD model achieved an AUC of 0.945 through triplet preprocessing and 3D hybrid Transformer, validating the synergistic advantages of deep feature engineering and multi-task learning. Gao et al.’s (16) generative network maintained an AUC of 0.912 in scenarios with missing data, demonstrating the adaptability of cross-modal completion technology to real-world data. Odusami et al.’s (14) pixel-level ViT fusion reached an AUC of 0.897 in single-modality MRI analysis, proving Transformer’s capability for high-resolution representation of imaging details. These results collectively indicate that innovations in model architecture (such as hybrid Transformer) and optimization of data fusion strategies are core pathways to improving diagnostic efficacy.

Although this study confirmed stable results (AUC fluctuation: 0.920–0.928) and no significant publication bias (Egger’s test, *p* = 0.217) through sensitivity analysis, the following limitations should be noted: First, only seven of the included studies use multicenter data, and single-center bias may limit the model’s performance in cross-cohort generalization (4, 6). Second, the efficacy difference between hybrid Transformer and pure Transformer models do not reach statistical significance (*p* = 0.068), indicating that the feature complementarity mechanism between traditional neural networks and Transformer requires further validation (11). Additionally, insufficient model interpretability remains a major obstacle to clinical application, as the black-box nature of attention mechanisms struggles to meet the transparency requirements of diagnostic decision-making (28).

Future research needs to focus on three major directions: First, promoting standardized integration of multicenter data and reducing the impact of data heterogeneity through technologies such as federated learning. Second, developing interpretability modules, such as introducing attention heatmaps to visualize brain region-pathology associations (23). Third, optimizing lightweight model design by borrowing the attention bottleneck mechanism proposed by Kadri et al. (19) to balance computational requirements and diagnostic accuracy. With the deep integration of Transformer technology with medical imaging and clinical data, such models are expected to become core tools for early and precise AD diagnosis, providing critical support for achieving the clinical goal of “early detection and early intervention.”

## 5 Conclusion

This study systematically evaluates the efficacy of Transformer-based multimodal fusion deep learning models in early AD diagnosis through meta-analysis. Results showed that these models achieved an overall AUC of 0.924 (95% CI: 0.912–0.936), significantly superior to traditional methods, confirming the deep modeling capability of Transformer’s self-attention mechanism for cross-modal data (e.g., MRI, PET, clinical indicators). Subgroup analyses reveal that trimodal and above fusion (AUC = 0.935 vs. bimodal = 0.908, *p* = 0.012), intermediate fusion strategy (feature-level fusion, AUC = 0.931), and multicenter data (AUC = 0.930 vs. single-center = 0.918, *p* = 0.046) significantly improved diagnostic efficacy, indicating that the depth of multi-source data integration, fusion stage selection, and data heterogeneity management are key influencing factors. In typical studies, Khan et al.’s (20) 3D hybrid Transformer model achieved an AUC of 0.945 in AD vs. MCI discrimination, Gao et al.’s (16) generative network maintained an AUC of 0.912 with missing data, and Odusami et al.’s (14, 15) single-modality ViT fusion reached an AUC of 0.897, respectively validating the models’ advantages in feature engineering, robustness, and imaging detail representation. Although sensitivity analysis shows stable results (AUC fluctuation: 0.920–0.928) and no significant publication bias (Egger’s test, *p* = 0.217), limitations such as a high proportion of single-center data and insufficient model interpretability were identified. Future research should focus on standardized multicenter data integration, development of interpretability modules (e.g., attention visualization), and lightweight design to promote clinical translation. In conclusion, Transformer-based multimodal fusion models provide highly effective tools for early AD diagnosis, with remarkable potential in dynamically modeling cross-modal associations. Technical innovations are urgently needed to address current bottlenecks and facilitate the clinical goal of “early diagnosis and early treatment” for AD.

## Data Availability

The original contributions presented in the study are included in the article/Supplementary material, further inquiries can be directed to the corresponding author.
